# Supplementary material for: Cryo-EM structures of mitochondrial ABC transporter ABCB10 in apo and biliverdin-bound form
Source: Nat Commun. 2023 Apr 11;14:2030. doi: 10.1038/s41467-023-37851-9 (PMC10090120; doi:10.1038/s41467-023-37851-9)
Supplement: Supplementary file 1 — Supplementary Information [file 41467_2023_37851_MOESM1_ESM.pdf]

Supplementary Information

Cryo-EM Structures of mitochondrial ABC transporter ABCB10 in apo and biliverdin-bound form

Sheng Cao[1#], Yihu Yang[1#], Lili He[2], Yumo Hang[2], Xiaodong Yan[1], Hui Shi[1], Jiaquan Wu[1], Zhuqing Ouyang[2*]

Affiliations:

[1]Wuxi Biortus Biosciences Co. Ltd., 6 Dongsheng Western Road, Jiangyin, Jiangsu 214437, China.

[2]Department of Pathogen Biology, School of Basic Medicine, Tongji Medical College, Huazhong University of Science and Technology, 13 Hangkong Road, Wuhan, Hubei province, 430030, China

[#]These authors contributed equally: Sheng Cao, Yihu Yang

[*]**Corresponding authors**:

Email: zhuqingouyang@hust.edu.cn

<u>Supplementary Information</u>

Supplementary Information file include Supplementary Figures 1–6.

Supplementary Figure 1: Characterization of ABCB10

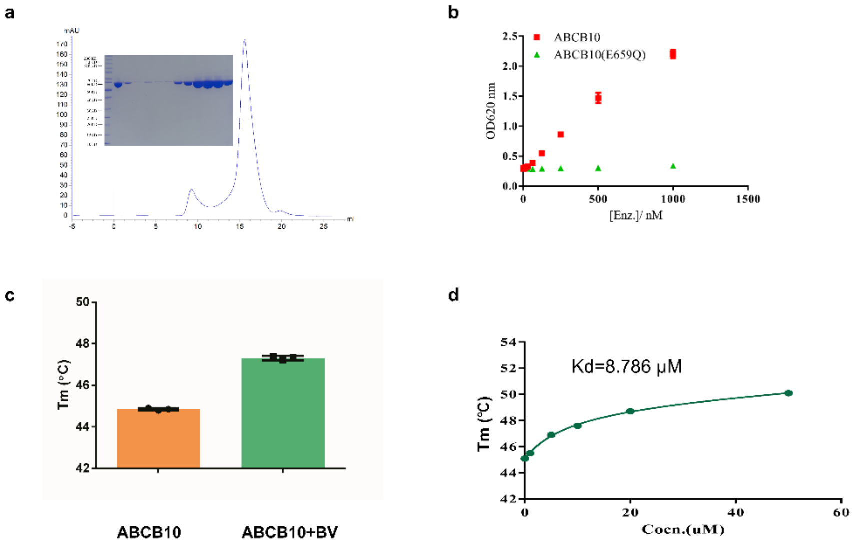

a. Size-exclusion profile of ABCB10 and SDS-PAGE analysis of eluted fractions. This experiment was repeated 3 times independently with similar results.
b. ATPase activity of ABCB10 WT and ATPase dead mutant (E659Q). ATPase activities are measured at varied concentration of ABCB10 WT or E659 mutant. Each data is presented as the means±s.d. of three independent assays (n=3).
c. BV increases the thermal stability of ABCB10. The melting temperatures (Tm) of ABCB10 are measured by performing thermofluor shift assays. Each data is presented as the means±s.d. of three independent assays (n=3).
d. BV binding to ABCB10. The melting temperatures (Tm) were measured in the presence of increasing BV concentrations.

Supplementary Figure 2: Cryo-EM data processing

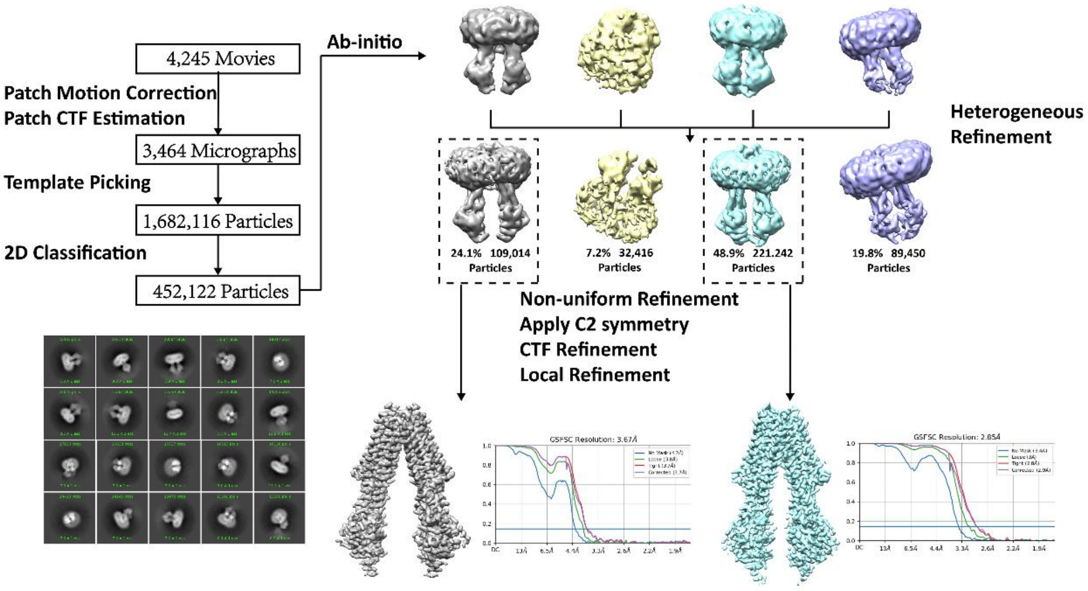

Representative 2D classifications and work-flow of cryo-EM data processing for ABCB10-apo and ABCB10-BV. 'Gold standard' FSC curve indicates overall nominal resolution at 3.67Å and 2.85Å using the FSC = 0.143 criterion.

Supplementary Figure 3: Cryo-EM maps and refined structures of ABCB10-apo

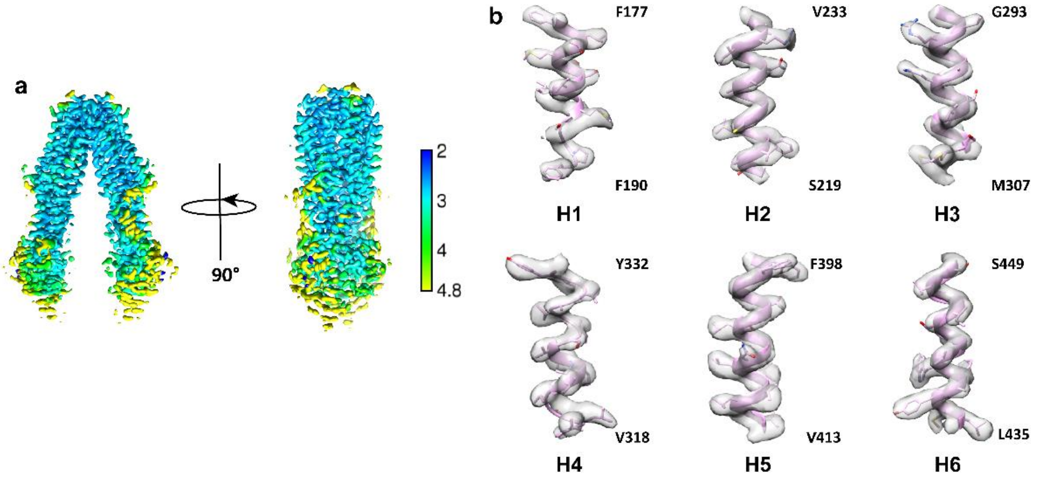

a. Local resolution estimation for ABCB10-apo.
b. Density maps and models for residues in transmembrane helixes near ligand binding pocket

Supplementary Figure 4: Cryo-EM maps and refined structures of ABCB10-BV

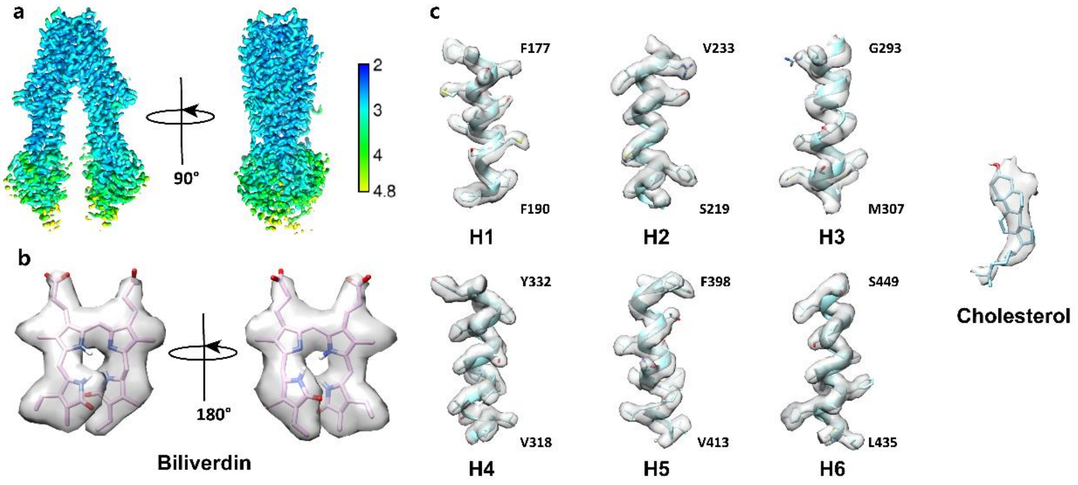

a. Local resolution estimation for ABCB10-BV.
b. Density and model fitting for biliverdin in two orientations.
c. Density maps and models for residues in transmembrane helixes near ligand binding pocket and cholesterol.

Supplementary Figure 5: Electrostatic surfaces for the translocation pathway of ABCB10-apo and ABCB10-BV

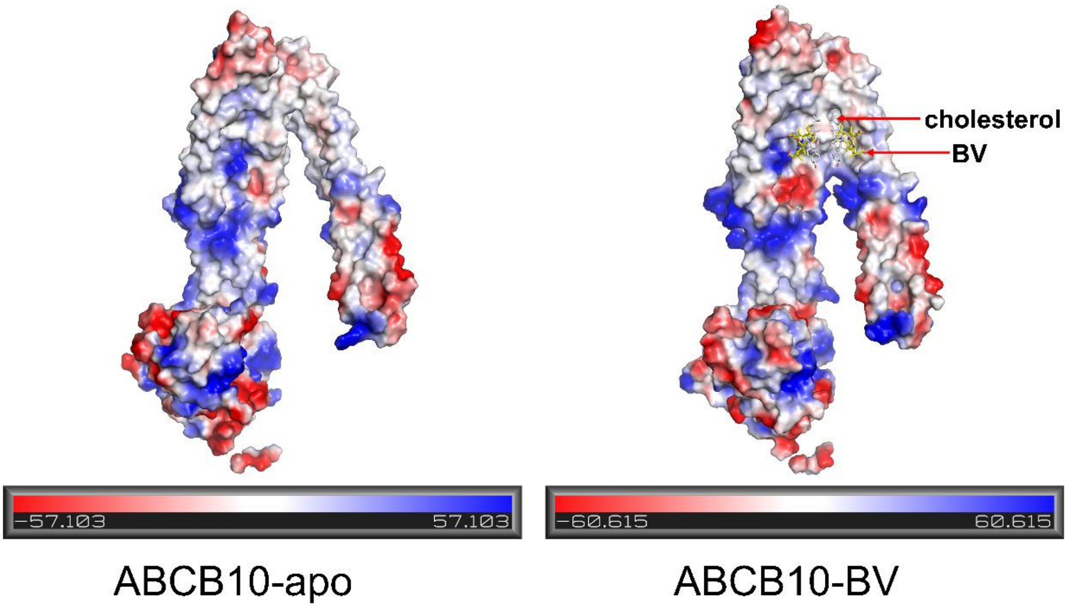

Electrostatic surfaces drawing of ABCB10-apo and ABCB10-BV showing the substrate translocation pathway with only one protomer shown for clarity. Red represents areas of negative electrostatic potential and blue areas represent positive electrostatic potential. BVs and cholesterols are indicated and shown in yellow and white sticks, respectively.

Supplementary Figure 6: Sequence alignment of eukaryotic ABCB10

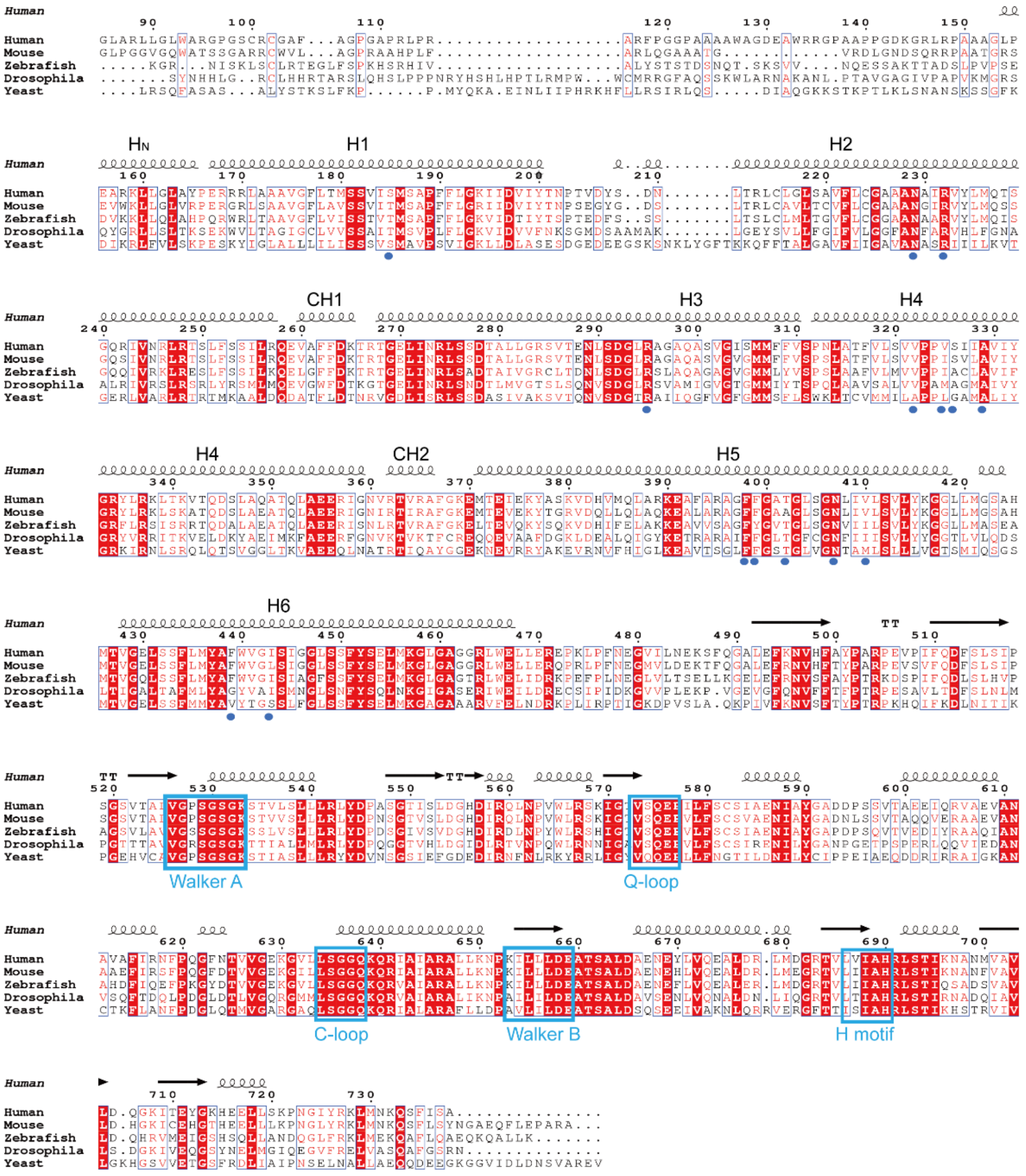

Sequence of human ABCB10 (Uniprot: Q9NRK6), mouse ABCB10 (Q9JI39), zebrafish ABCB10 (F1Q5K6), *Drosophila* CG3156 (Q8SWW9) and yeast multidrug resistance-like 1 (P33310) are shown. The multiple-sequence alignment of eukaryotic ABCB10 was created by Clustal w2. The secondary structure assignments based on the cryo-EM structure of ABCB10-BV are indicated on the first line of the alignment. Residues involved in BV interaction are indicated by blue dots. Conserved motifs in NBD are boxed and indicated.
